# Fertility Behavior and Depression Among Women: Evidence From China

**DOI:** 10.3389/fpsyg.2020.565508

**Published:** 2020-11-13

**Authors:** Hualei Yang, Xiaodong Zheng, Ruyin Zhou, Zheng Shen, Xinyu Huang

**Affiliations:** ^1^School of Public Administration, Zhongnan University of Economics and Law, Wuhan, China; ^2^School of Economics, Zhejiang Gongshang University, Hangzhou, China; ^3^College of Economics and Management, China Agricultural University, Beijing, China; ^4^School of Economics and Management, Zhejiang A&F University, Hangzhou, China

**Keywords:** fertility, depression, women, China, China Labor-Force Dynamics Survey

## Abstract

Using data from the China Labor-Force Dynamic Survey, this study employed logistic regressions to investigate the association between fertility behavior and depression among Chinese women. The empirical results show that in China, women having children were significantly less likely to have depressive symptoms (*OR* = 0.651) compared to childless women. We also found a U-shaped relationship between fertility levels and depression in women. The results were robust to using the propensity score matching approach to address the sample selection problem. Further, our heterogeneity analysis demonstrated that the negative relationship between fertility level and depression was more significant among women who were in their 30s, lived in urban areas, and lived in high-income households. Compared to having male children (boys) (*OR* = 0.874), having female children (girls) (*OR* = 0.795) was more significantly associated with fewer depressive symptoms among women. In the meantime, we did not find a significant relationship between the childbearing period and depression. The paper discussed possible reasons for our findings and policy implications from the perspectives of the government, society, and family.

## Introduction

Depressive disorders have become an increasingly important health risk worldwide, seriously affecting people’s quality of life and their happiness. Such disorders are also more prevalent among women (Badger et al., 2004; Sadat et al., 2014). Considering that women face the dual pressure of household work and career development (Simon, 1992; Cunningham, 2008), the unique factors that affect depression in women have attracted increasing attention from the academic community and the public. Fertility behavior is one of such factor.

There is no consensus regarding the relationship between fertility behavior and depression among women. On the one hand, many previous studies found that both having children as well as having a higher number of children could increase depression levels among women (Catherine and Ross, 1996; Dew and Wilcox, 2011; Shi, 2015; Roeters et al., 2016). Sudha et al. (2006) demonstrated that the number of children and pregnancies significantly increased the incidence of depressive symptoms among African-American women, while their counterparts who had never given birth showed fewer depressive symptoms. On the other hand, studies have also found that fertility behavior did not significantly increase or reduce depression in women (Savolainen et al., 2001; Keizer et al., 2010; Meier et al., 2016). For instance, Grundy and Read (2015) showed that, compared with two children, childlessness or having one child was associated with more severe depressive symptoms among older women.

Theoretically, fertility behavior may adversely affect women’s depression level by influencing their physical health (Beral, 1985; Grundy and Read, 2015); increasing household work (Dew and Wilcox, 2011; Roeters et al., 2016); and decreasing wages, interpersonal communications, and social activities (Killewald, 2013; Roeters et al., 2016). However, having children may also improve women’s well-being as they expect to receive emotional and economic support from their children. Gibney et al. (2017) used data from the Survey of Health, Ageing, and Retirement in Europe (SHARE) and found that childlessness was significantly associated with depressive symptoms among older women because of the lack of financial and emotional support. The direction and extent of the relationship between fertility and depression may lie in the quantity and quality of children and could be influenced by other important factors such as a woman’s age, time and period of having children, and social context.

As past research, findings on the relationship between fertility behavior and depression among women are mixed, and the studies are mostly limited to developed countries. More in-depth studies are needed in the context of developing countries such as China. Due to its one-child policy, China’s fertility rate has been low since the 1980s. However, even after the introduction of the universal two-child policy in 2015, the fertility rate of the country has not improved significantly and has, instead, experienced a slight decline. In 2019, China had 14.65 million births, a birth rate of 10.48 per thousand people, and a death rate of 7.14 per thousand. The natural population growth rate was 3.34 per thousand, the lowest since 1952. Under these circumstances, the relationship between fertility behavior and the mental health of women can improve our understanding of the effects of fertility policies in China, which also has implications for the rest of the world.

This study aims to investigate the association between fertility behavior and depressive symptoms among Chinese women. Using data from the China Labor-Force Dynamics Survey (CLDS), this study employed logistic regressions to examine the relationship between the number of children and depression. Considering the potential sample selection problem, the propensity score matching (PSM) approach was used to determine the robustness of the research findings. Furthermore, we analyzed the heterogeneity of the relationship considering women’s age, residence area, and household income. Finally, we explored the associations of the child’s gender and the childbearing period with depression in young and middle-aged Chinese women. This paper contributes toward testing the applicability of existing theoretical inferences and empirical research to Chinese samples and expanding the universality of existing research findings.

This paper proceeds as follows. Section “Materials and Methods” describes the data, measures, and empirical strategy. Section “Results” presents the regression results with robustness checks. Finally, Section “Conclusion” concludes the paper with a discussion of the policy implications.

## Materials and Methods

### Data

The data used in this was drawn from the 2016 CLDS. The CLDS is a labor dynamic survey project in mainland China that has been conducted by the Social Science Survey Center of Sun Yat-sen University since 2012. The survey aims to depict the current situation and changes in the Chinese labor force. The CLDS survey object is the labor force in the sample households. In terms of the sampling method, the survey adopts a multi-stage and multi-level probability sampling method proportional to the size (PPS) of the labor force, and the data are nationally representative. In 2016, 11,631 households from 158 cities in 29 provinces in mainland China were surveyed. Two key sample restrictions were applied to facilitate our analysis. First, since women aged 52 years or below were asked questions regarding their history of pregnancy and fertility, we restricted our sample to married female respondents who provided such information. Second, to ensure that the results are not affected by factors other than normal fertility, women who became pregnant but failed to give birth normally or whose children died prematurely after birth were excluded. Finally, our sample consisted of 5,643 Chinese women aged 16–52 years.

### Measures

#### Depressive Symptoms

We measured the depression level among women using the Center for Epidemiologic Studies Depression Scale (CES-D) developed by Radloff in 1977. Because of its high reliability and validity, this self-report questionnaire is widely used to measure the severity of depressive symptoms in the general population, including adolescents, adults, and older individuals (Radloff, 1977; Shafer, 2006). Evidence shows that the CES-D is also a valid, reliable, sensitive, and responsive instrument for screening and monitoring depressive symptoms in Chinese adults (Lai, 1995; Chin et al., 2015). The CES-D consists of 20 questions regarding different aspects of depressive symptoms experienced in the past week, including questions about depressive affect, positive affect, somatic symptoms, and interpersonal problems (see Table A1). Respondents rate the frequency of occurrence of each symptom (question) on a 4-point Likert scale (*rarely or none of the time (<1 day) = 0; some or a little of the time (1–2 days) = 1; occasionally or a moderate amount of the time (3–4 days) = 2*; and *most or all of the time (5–7 days) = 4*). A total score ranging from 0 to 60 is obtained after adding up the scores of each item. Higher scores indicate more severe depression. The CES-D also provides a cutoff score (16 or higher) to identify individuals at risk for clinical depression, with high internal consistency (Lewinsohn et al., 1997; Gatz et al., 2005). Accordingly, we generated a binary variable to measure depressive symptoms among women, which was scored as 1 if the CES-D score was 16 or higher; otherwise, it was scored as 0.

#### Fertility Behavior

In the CLDS, women aged 52 years or below provided their historical information on fertility behaviors. The fertility level was determined based on responses to the question “How many children have you had?” We also generated a binary variable to measure whether the respondent had children (*Yes = 1*). Additionally, we selected variables related to fertility, including having male children (boys) (*Yes = 1*), number of boys, having female children (girls) (*Yes = 1*), number of girls, age at birth of first child (*below 20 = 0*, *20–24 = 1*, *25–29 = 2*, *30 or above = 3*), and childbearing years (*1 year = 0*, *2–4 years = 1*, *5 years or above = 2*), to further investigate the association of children’s gender and women’s childbearing age with depression in women. This helped us to better understand the overall picture of the relationship between fertility behavior and the mental health of women. The age of women’s first childbearing and the number of childbearing years were obtained from the questions “How old were you when your first child was born?” and “How old were you when your last child was born?”

#### Control Variables

Past research has shown that, among women, various factors are associated with mental health (Shi, 2015). To control for the observed characteristics of women and their families, the control variables in the present study included women’s *age*, *education level (years of education)*, *religious belief (yes = 1)*, *household registration (hukou) status (1 = urban)*, *self-reported health (1 = very healthy*, *2 = healthy*, *3 = average*, *4 = unhealthy*, *5 = very unhealthy)*, *regular physical exercise (yes = 1)*, *neighborhood assistance (neighbors and other residents often help each other = 1)*, *subjective socioeconomic status (ranging from 1 (lowest) to 10 (highest))*, and *household income (logarithm).* Table 1 shows the descriptive statistics of the main variables.

**TABLE 1:** Descriptive statistics.

Variable	Symbol	Mean	SD	Min	Max
Depressive symptoms	*depre*	0.174	0.379	0	1
Have children	*child*	0.811	0.391	0	1
Fertility level	*fert*	1.428	1.005	0	8
Age	*age*	39.754	8.765	16	52
Education	*edu*	7.563	3.809	0	21
Religion	*reli*	0.152	0.359	0	1
Household registration status	*ident*	0.181	0.385	0	1
Self-reported health	*slfhlth*	3.686	0.954	1	5
Physical exercise	*exer*	0.280	0.449	0	1
Neighborhood assistance	*neig*	0.803	0.398	0	1
Subjective socioeconomic status	*ses*	4.420	1.658	1	10
Household income (log)	*hholdinc*	10.505	1.462	0	14.078

### Statistical Analysis

Since the dependent variable (whether the respondents had depressive symptoms) in our data analysis was binary, our study used logistic regression and reported odds ratios (ORs) to investigate the association between fertility behavior and depression in Chinese women. For variables of fertility behavior, we first checked whether the respondent women had children (*child*) to examine the general association of having children with depression. Then, we used the number of children (*fert*) and its quadratic term (*fert_sqr*) to explore the linear and nonlinear relationship between fertility level and depressive symptoms. Additionally, to check the robustness of our findings, we employed the PSM approach to balance respondents with different fertility levels. Specifically, we first used control variables that might influence the probability of having children and the number of children, and conducted logistic regressions to estimate the propensity scores. These scores were used to match the respondents in the treatment and control groups within the common support region. We then conducted a balance check on the covariates. Finally, matched samples with balanced characteristics were used to estimate the differences in the probability of having depressive symptoms between the two groups as treatment effects of having children or of the fertility level. The statistical analysis in this study was conducted using Stata version 15.0 (StataCorp, Texas, United States).

## Results

### Fertility Behavior and Depression Among Chinese Women

Table 2 reports the estimation results of the relationship between fertility behavior and depressive symptoms among Chinese women by logistic regression. As shown in Models 1 and 2, after controlling for relevant variables that might correlate with depression in women, having children (*OR* = 0.651) and the number of children (*OR* = 0.910) were significantly and negatively associated with the probability of being depressed, indicating that women who had children experienced less severe depressive symptoms. Based on Model 2, Model 3 added the quadratic term of fertility (*fert_sqr*) to explore the nonlinear relationship between fertility level and depression in women. As the regression results show, the quadratic term of fertility was positively significant, suggesting a U-shaped association between the number of children and depressive symptoms. In other words, having children does not always predict fewer depressive symptoms for women. With the increase in the number of children, after a cutoff, women may experience more severe depression. We also found that education, self-reported health, physical exercise, neighborhood assistance, subjective socioeconomic status, and household income were negatively associated with depression among women.

**TABLE 2:** Fertility behavior and depressive symptoms among women—logistic regression.

Variable	Model 1		Model 2		Model 2	
			
	OR	95%CI	OR	95% CI	OR	95% CI
*child*	0.651***	[0.546, 0.778]				
*fert*			0.910**	[0.847, 0.979]	0.749***	[0.644,0.873]
*fert_sqr*					1.057***	[1.018, 1.097]
*age*	0.991*	[0.982, 1.001]	0.995	[0.986, 1.004]	0.993	[0.984, 1.002]
*edu*	0.974**	[0.951, 0.998]	0.973**	[0.950, 0.997]	0.974**	[0.951, 0.998]
*reli*	1.150	[0.946, 1.399]	1.177	[0.969, 1.430]	1.165	[0.958, 1.415]
*ident*	1.157	[0.924, 1.448]	1.134	[0.905, 1.420]	1.127	[0.899, 1.411]
*slfhlth*	0.582***	[0.538, 0.631]	0.581***	[0.536, 0.629]	0.583***	[0.538, 0.631]
*exer*	0.841*	[0.706, 1.002]	0.832**	[0.699, 0.991]	0.840*	[0.705, 1.000]
*neig*	0.828**	[0.694, 0.988]	0.833**	[0.699, 0.993]	0.834**	[0.699, 0.994]
*ses*	0.877***	[0.840, 0.917]	0.880***	[0.843, 0.920]	0.877***	[0.839, 0.916]
*hhldinc*	0.950**	[0.907, 0.995]	0.944**	[0.902, 0.989]	0.949**	[0.906, 0.993]
*N*	5,643		5,643		5,643	

OR, odds ratio. 95% confidence intervals in brackets. *p < 0.1, **p < 0.05, ***p < 0.01.

### Heterogeneity Analysis

We further explored the heterogeneity of the relationship between the number of children and depressive symptoms among women by subgroup estimation and comparison, which are shown in Tables 3–5. Table 3 reports the age group, urban–rural, and household income group differences in the association between fertility and depression. First, we divided our sample into three age groups: up to 30 years of age, 30–40 years of age, and 40–52 years old. Models 1–3 report the estimates of the three subsamples. The results show that, compared to relatively young (Age ≤ 30 years) and older (40 < Age ≤ 52 years) women, the relationship between fertility level and depression was more significant for women aged 30–40 years (*p* < 0.05). This implies that age group differences in the negative association between the number of children and depression in women. Second, the total sample was divided into urban and rural subgroups, the results of which are shown in Models 4 and 5. The association between fertility and depression is larger for urban women (*OR* = 0.727) than for rural women (*OR* = 0.926), indicating that, as compared to rural women, urban women with more children are less likely to have depression symptoms. Third, the total sample was divided into two groups according to the median household income, the estimates of which are reported in Models 6 and 7. The results demonstrated that fertility level was linked to fewer depressive symptoms among women who lived in high-income households (OR = 0.882) than those who lived in low-income households (*OR* = 0.922).

**TABLE 3:** Estimates of heterogeneity analysis: age groups, urban and rural, and household income groups.

Variable	Age groups			Urban and rural		Household income groups	
			
	Model 1	Model 2	Model 3	Model 4	Model 5	Model 6	Model 7
	Age ≤ 30	30 < Age ≤ 40	40 < Age ≤ 52	Urban	Rural	Above the median	Below the median
*fert*	0.855	0.812**	0.965	0.727**	0.926**	0.882*	0.922*
	[0.684, 1.068]	[0.687, 0.960]	[0.875, 1.064]	[0.553, 0.956]	[0.859, 0.999]	[0.776, 1.002]	[0.844, 1.006]
*age*	0.996	0.996	1.000	0.970**	0.999	0.990	1.000
	[0.939, 1.057]	[0.953, 1.040]	[0.967, 1.035]	[0.948, 0.994]	[0.989, 1.009]	[0.976, 1.005]	[0.988, 1.012]
*edu*	0.957	0.994	0.959**	0.959	0.974*	1.009	0.951***
	[0.899, 1.019]	[0.951, 1.039]	[0.928, 0.992]	[0.904, 1.018]	[0.949, 1.001]	[0.971, 1.049]	[0.922, 0.982]
*reli*	1.061	1.190	1.269*	1.499	1.150	1.250	1.187
	[0.691, 1.629]	[0.817, 1.735]	[0.966, 1.667]	[0.896, 2.507]	[0.931, 1.421]	[0.928, 1.685]	[0.915, 1.540]
*ident*	1.405	1.400*	0.905	–	–	1.118	1.096
	[0.822, 2.401]	[0.942, 2.080]	[0.651, 1.258]	–	–	[0.825, 1.517]	[0.768, 1.564]
*slfhlth*	0.615***	0.605***	0.564***	0.782**	0.557***	0.538***	0.614***
	[0.495, 0.763]	[0.518, 0.706]	[0.509, 0.626]	[0.630, 0.971]	[0.511, 0.607]	[0.472, 0.613]	[0.555, 0.679]
*exer*	0.666*	0.706**	0.996	0.618**	0.918	0.704***	0.985
	[0.423, 1.047]	[0.507, 0.982]	[0.787, 1.261]	[0.427, 0.894]	[0.754, 1.118]	[0.547, 0.905]	[0.771, 1.257]
*neig*	0.825	1.015	0.750**	0.767	0.846*	0.810	0.869
	[0.567, 1.201]	[0.724, 1.422]	[0.584, 0.963]	[0.521, 1.129]	[0.694, 1.032]	[0.627, 1.047]	[0.681, 1.110]
*ses*	0.881**	0.889***	0.870***	0.874**	0.878***	0.876***	0.884***
	[0.788, 0.984]	[0.819, 0.965]	[0.820, 0.923]	[0.782, 0.976]	[0.837, 0.921]	[0.817, 0.940]	[0.835, 0.935]
*hhldinc*	1.085	0.906**	0.929**	0.984	0.936***	0.969	0.975
	[0.936, 1.258]	[0.827, 0.993]	[0.877, 0.984]	[0.853, 1.135]	[0.892, 0.983]	[0.777, 1.207]	[0.921, 1.031]
*N*	1,107	1,708	2,828	1,018	4,625	2,804	2,839

OR, odds ratio. 95% confidence intervals in brackets. *p < 0.1, **p < 0.05, ***p < 0.01.

**TABLE 4:** Estimates of heterogeneity analysis: gender of the child.

	Model 1	Model 2	Model 3	Model 4
*have boys*	0.874*			
	[0.753, 1.015]			
*number of boys*		0.975		
		[0.879, 1.080]		
*have girls*			0.795***	
			[0.688, 0.919]	
*number of girls*				0.872***
				[0.793, 0.959]
*age*	0.996	0.997	0.995	0.996
	[0.987, 1.005]	[0.987, 1.006]	[0.986, 1.004]	[0.986, 1.005]
*edu*	0.976*	0.978*	0.975**	0.975**
	[0.953, 1.000]	[0.954, 1.001]	[0.952, 0.999]	[0.952, 0.998]
*reli*	1.169	1.178*	1.175	1.179*
	[0.962, 1.421]	[0.970, 1.431]	[0.967, 1.427]	[0.970, 1.432]
*ident*	1.145	1.157	1.146	1.144
	[0.915, 1.435]	[0.924, 1.449]	[0.915, 1.435]	[0.914, 1.433]
*slfhlth*	0.583***	0.583***	0.583***	0.582***
	[0.538, 0.631]	[0.538, 0.631]	[0.539, 0.631]	[0.538, 0.630]
*exer*	0.838**	0.841*	0.841*	0.838**
	[0.704, 0.998]	[0.706, 1.001]	[0.706, 1.002]	[0.703, 0.997]
*neig*	0.834**	0.834**	0.832**	0.833**
	[0.699, 0.994]	[0.699, 0.994]	[0.698, 0.992]	[0.699, 0.993]
*ses*	0.880***	0.880***	0.880***	0.881***
	[0.842, 0.919]	[0.843, 0.920]	[0.842, 0.919]	[0.843, 0.921]
*hhldinc*	0.946**	0.945**	0.943**	0.942**
	[0.904, 0.990]	[0.902, 0.989]	[0.901, 0.988]	[0.900, 0.986]
*N*	5,643	5,643	5,643	5,643

OR, odds ratio. 95% confidence intervals in brackets. *p < 0.1, **p < 0.05, ***p < 0.01.

**TABLE 5:** Estimates of heterogeneity analysis: childbearing period.

	Model 1	Model 2	Model 3

Age at birth of first child			
20–24	1.243		1.263
	[0.875, 1.766]		[0.889, 1.796]
25–29	1.104		1.131
	[0.759, 1.605]		[0.775, 1.651]
≥ 30	1.143		1.191
	[0.687, 1.901]		[0.711, 1.994]

**Childbearing years**			

2–4		1.015	1.010
		[0.815, 1.265]	[0.801, 1.273]
≥ 5		1.164	1.167
		[0.944, 1.434]	[0.933, 1.461]
*age*	0.997	0.996	0.996
	[0.984, 1.009]	[0.984, 1.007]	[0.983, 1.008]
*edu*	0.988	0.987	0.990
	[0.959, 1.018]	[0.959, 1.015]	[0.961, 1.020]
*reli*	1.181	1.111	1.172
	[0.928,1.502]	[0.884, 1.396]	[0.921, 1.492]
*ident*	1.135	1.106	1.160
	[0.862, 1.494]	[0.844, 1.450]	[0.876, 1.536]
*slfhlth*	0.583***	0.586***	0.582***
	[0.530, 0.642]	[0.535, 0.641]	[0.529, 0.641]
*exer*	0.781**	0.794**	0.783**
	[0.631, 0.965]	[0.647, 0.973]	[0.633, 0.969]
*neig*	0.835*	0.846*	0.832*
	[0.679, 1.027]	[0.693, 1.032]	[0.676, 1.023]
*ses*	0.885***	0.883***	0.885***
	[0.840, 0.932]	[0.840, 0.928]	[0.840, 0.933]
*hhldinc*	0.944**	0.950*	0.947*
	[0.892, 0.999]	[0.899, 1.002]	[0.895, 1.002]
*N*	4,199	4,199	4,199

OR, odds ratio. 95% confidence intervals in brackets. *p < 0.1, **p < 0.05, ***p < 0.01.

Table 4 presents the estimates of the heterogeneity analysis regarding gender of the child. Models 1–4 show that both having boys (or the number of boys) and having girls (or the number of girls) were negatively associated with depression in women. Women who had girls (*OR* = 0.795) were less likely to have depression symptoms than those who had boys (*OR* = 0.874). This suggests that having girls is linked to lower levels of psychological stress among Chinese women. Table 5 reports the estimates of the association between childbearing period and depression in women. As shown in Models 1–3, although the increases in the age at birth of first child and childbearing years were likely to increase the OR of being depressed, we did not find a statistically significant relationship between the childbearing period and depressive symptoms among Chinese women.

### Robustness Checks

**FIGURE 1:**
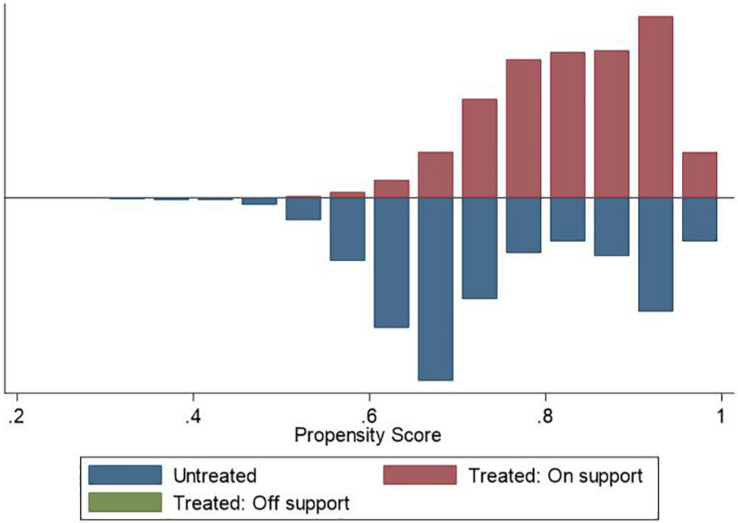
Density distribution of treated and untreated observations (Fertility ≥ one child).

**FIGURE 2:**
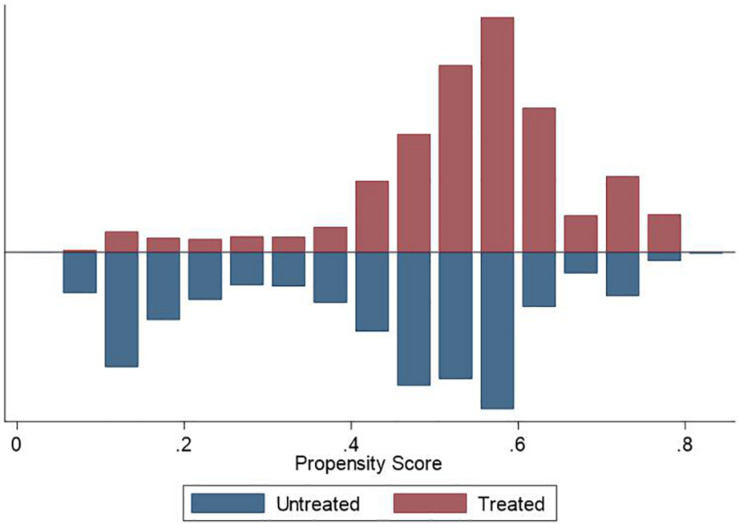
Density distribution of treated and untreated observations (Fertility ≥ two children).

**FIGURE 3:**
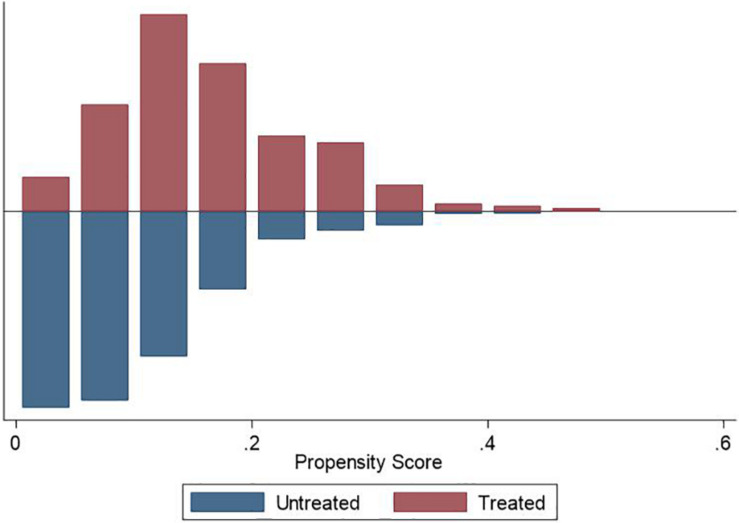
Density distribution of treated and untreated observations (Fertility ≥ three children).

We used the PSM approach to check the robustness of our main findings. We used different matching techniques, including nearest neighbor matching (1:1 and 1:3), radius matching, and kernel matching, to ensure the consistency of the estimation results. Figures 1–3 demonstrate that the kernel density distribution of the treated and untreated groups was relatively close after matching. This suggests a good balance between the treatment and control groups and that the matching estimates have high validity. As shown in Table 6, the estimates are almost consistent among the three matching approaches. Compared to childless women, women who had children experienced significantly fewer depressive symptoms. However, there was no significant difference in depression between women who had less than two children and those who had two or more children. Similarly, when comparing women who had less than three children to those who had at least three children, we did not find a significant difference in depressive symptoms. Overall, PSM estimates also suggest that having children was negatively associated with depression among women. However, more children did not necessarily represent lower levels of depression, indicating a U-shaped relationship between fertility level and depressive symptoms among women. Therefore, our main findings are still robust after addressing the sample selection problem.

**TABLE 6:** Estimates of propensity score matching on the association between fertility behavior and depressive symptoms among women.

	Nearest neighbor matching (1:1)	Nearest neighbor matching (1:3)	Radius matching (*k* = 0.05)	Kernel matching
				
	(1)	(2)	(3)	(4)
ATT (Fertility ≥ one child)	0.917***	0.918***	0.929***	0.932***
	[0.875, 0.961]	[0.881, 0.956]	[0.895, 0.964]	[0.9, 0.966]
ATT (Fertility ≥ two children)	0.982	0.981	0.983	0.988
	[0.954, 1.011]	[0.956, 1.007]	[0.962, 1.005]	[0.967, 1.01]
ATT (Fertility ≥ three children)	0.998	0.995	0.995	0.996
	[0.95, 1.048]	[0.957, 1.035]	[0.961, 1.031]	[0.961, 1.032]

OR, odds ratio. 95% confidence intervals in brackets. *p < 0.1, **p < 0.05, ***p < 0.01.

## Discussion

Based on the CLDS data, this study employed an instrumental variable estimation approach to investigate the association between the number of children and depression levels among Chinese women. The empirical results suggest that having children was significantly associated with a lower likelihood of having depressive symptoms among Chinese women compared with childless women. We also found a U-shaped relationship between fertility level and depression in women. To ensure the robustness of our results, we used the PSM approach to address the sample selection problem and found that our findings are still robust. Further heterogeneity analysis demonstrated that the negative relationship between fertility level and depression was more significant for women who were in their 30s, lived in urban areas, and lived in high-income households. Compared to having boys, having girls was more significantly associated with fewer depressive symptoms among Chinese women. Meanwhile, we did not find a significant relationship between the childbearing period and depression in Chinese women.

Our results suggest that women who had children were less likely to be depressed compared to women without children, which can be explained by the health benefits of parenthood. First, from the physiological perspective, pregnancy, childbirth, and lactation may affect women’s physical health. Evidence has shown that compared with women who have not had children, women who have had children were less likely to die from breast, ovarian, and endometrial cancers (Beral, 1985). Second, having children may provide parents with a greater incentive to follow a healthier lifestyle (Umberson et al., 2010). It is suggested that, compared with childless older people, older couples with children had healthier lifestyles (Kendig et al., 2007). Third, parenthood may increase social participation and social support. Many studies have indicated that parenthood may enhance community participation (Furstenberg, 2005; Offer and Schneider, 2007). Moreover, social support from children may bring health benefits to parents in later stages of life (Antonucci et al., 2003; Barefoot et al., 2005; Krause, 2007).

In addition, we found a U-shaped relationship between the number of children and depression, which means that having more children was not necessarily associated with more happiness. There are four main reasons for this. First, with more children, women may work less or not work at all outside the home, limiting their career progression. Their interpersonal communication would reduce and they would receive less social support because of their constant involvement in looking after children, which may easily lead to depression. Second, having more children may cause more physical problems and subsequently influence the mental or depression status of parents. Grundy and Tomassini (2005) suggested that having five or more children was associated with a higher risk of long-term disease in later life. They also found that the number of children had a positive correlation with the mortality risk of ischemic heart disease, coronary heart disease, circulatory system disease, cervical cancer, and other diseases, and a negative correlation with death caused by ovarian cancer, uterine cancer, breast cancer, and other diseases. Third, with the increase in the number of children, the adverse health effects due to stress in rearing children may exceed the health benefits expected from children because of the weakening role of children as economic contributors (Grundy and Kravdal, 2010). Fourth, an increase in the number of children may result in declining quality of each child, and the children may shirk their responsibility to support their parents in later life. According to the “Substitution Model for Quantity and Quality of Children” proposed by Becker (1992), when resources such as family income are constrained, the medical and educational resources allocated to each child are reduced, leading to lower average quality of each child. It is suggested that children with more years of education can provide more material support and improve the quality of life and health status of their elderly parents (Steelman et al., 2002). Moreover, with more members in the family, the family relationship can be complicated and prone to problems such as intergenerational conflicts and economic conflicts, which is not beneficial to the quality of life of parents.

In the heterogeneity analysis, the results show that the association between fertility behavior and depression differed based on women’s age, local characteristics, and household income. Compared to relatively young and older women, women aged 30–40 years with more children were less likely to have depression. This result needs to be explained in the special context of China. Influenced by Confucianism and a filial piety culture that values family continuity, married women are under significant pressure to fulfill their family responsibilities (Chen, 2018). In China, at the age of 30 years, most women are married, with the corresponding numbers being 82.0% in the urban area and 94.6% in the rural area (He et al., 2019). For married women without children, childbearing expectations from their families may lead to increased stress and consequent depressive symptoms (Logan et al., 2019). Moreover, we observed that fertility level was linked to fewer depressive symptoms among women who lived in urban areas and those who lived in high-income households. Owing to higher living costs in urban areas, urban women without children may face greater economic pressure in the absence of economic support from children in later life, and may suffer from poorer mental health due to lack of sufficient emotional support. We also found that for women in high-income households, having more children was associated with fewer depressive symptoms. One possible explanation could be that fertility behavior could relieve the pressure of childbearing expectations from their families without imposing a heavy economic burden.

The results also show that women having girls were linked to a lower level of psychological stress than those having boys, which is consistent with the existing research (Grundy and Read, 2015). Older people with daughters have an increased probability of regular social contact and receiving help when needed (Grundy and Read, 2015). However, the findings did not support the traditional Chinese view of “bringing up sons to support parents in their old age.” There are four possible reasons for this. First, even if having a son brings social affirmation in the traditional Chinese culture, where preference is accorded to sons, raising a son requires more economic and care resources, which increases the economic and psychological burden on parents. Second, the improvement of women’s status and the decrease in the number of children in the family have encouraged daughters to provide more support for the elderly, weakening the role of sons in providing old-age support. Third, women’s advantages in family care have significantly increased the role of daughters in taking care of their aged parents. Fourth, the progress of agricultural technology and production tools and the increase in the proportion of non-agricultural income in rural families’ income sources have weakened the dependence of boys on their fathers. Although our study did not find a significant relationship between childbearing period and depression in women in the Chinese social context, some previous studies have indicated that giving birth too early or too late could increase the depressive symptoms among women. For example, Deal and Holt (1998) found that women aged 15–17 years were more than twice as likely as their adult counterparts to suffer from depression after giving birth. John and Ross (2002) suggested that when a woman has her first child at the age of 30 years, the risk of depression would be minimum. Due to data restriction, we are not able to empirically explore the reason behind the above difference. Meanwhile, although we employed the PSM approach to check the robustness of the results, researchers should still be cautious in making generalizations (e.g., causal effects) from our findings because of other potential hidden bias. Therefore, more relevant studies are needed to further investigate the relationship between fertility behavior and depression among women in different contexts.

In summary, the association between fertility behavior and depression is complex because fertility behavior has both positive and negative effects on the physical and mental health of Chinese women. The benefits of having children include lower risks of some diseases, healthier lifestyles, and increased social participation and support. However, women with more children may suffer from more physical problems and the declining quality of each child, and are more likely to have depressive symptoms. China’s population aging has currently plateaued. To activate the labor market and promote the sustainable and high-quality development of the country, there is an urgent need to optimize the population structure and encourage people to have more children. Accordingly, China has actively adjusted its fertility policy. However, while liberalizing fertility and encouraging more births, policymakers should also pay attention to the potential impact of changes in fertility behavior on the welfare of individuals and families, which should be used as the basis for the further development of fertility policy. Our findings could serve as a reference to relevant research in East Asian countries that have similar family systems and gender schema influenced by Confucian ideology and culture.

## Conclusion

This study empirically tested the association between fertility behavior and depression symptoms among Chinese women. The results indicate that having children was significantly associated with a lower likelihood of having depressive symptoms. However, with an increase in the number of children after a cutoff, women may experience more severe depression. Further analysis showed that the association between fertility and depression could be discussed in consideration of the differences in women’s age, local characteristics, household income, and gender of the child. In the context of the low fertility levels in China, the government, society, and families need to work together to build a fertility-friendly environment to reduce the depressive symptoms associated with fertility. At the government level, the focus should be on guiding the provision of preschool childcare services, reducing the costs of raising a child, expanding the scope and quality of childbirth-related services, and improving the protection of the rights and interests of women of childbearing age. The government should attempt to design tax policies favoring childbirth. At the social level, we need to cultivate a good culture for multi-child growth, encourage companies to provide fair career opportunities to multi-child families and undertake appropriate social responsibilities, and enhance the ability of social organizations to service the various needs of families with multiple children. At the family level, the implementation of family-based development plans should be encouraged. In terms of childcare, the participation of fathers needs to be strengthened, and grandparents can also participate appropriately.

## Data Availability Statement

The raw data supporting the conclusions of this article will be made available by the authors, without undue reservation, to any qualified researcher.

## Ethics Statement

Ethical review and approval was not required for the study on human participants in accordance with the local legislation and institutional requirements. Written informed consent from the participants was not required to participate in this study in accordance with the national legislation and the institutional requirements.

## Author Contributions

HY and XZ: conceptualization. HY and XH: methodology. XZ, RZ, and ZS: validation. HY, XZ, and RZ: formal analyses. HY, ZS, and XH: investigation. HY, XZ, RZ, and XH: writing – original draft preparation. All authors contributed to the article and approved the submitted version.

## Conflict of Interest

The authors declare that the research was conducted in the absence of any commercial or financial relationships that could be construed as a potential conflict of interest.

## Appendix

**TABLE A1:** Center for Epidemiologic Studies Short Depression Scale (CES−D 20).

Items:	Rarely or none of the time (< 1 day)	Some or a little of the time (1–2 days)	Occasionally or a moderate amount of the time (3–4 days)	Most or all of the time (5–7 days)
1. I was bothered by things that don’t usually bother me				
2. I did not feel like eating; my appetite was poor				
3. I felt that I could not shake off the blues even with the help of my family or friends				
4. I felt that I was just as good as other people				
5. I had trouble keeping my mind on what I was doing				
6. I felt depressed				
7. I felt everything I did was an effort				
8. I felt hopeful about the future				
9. I thought my life had been a failure				
10. I felt fearful				
11. My sleep was restless				
12. I was happy				
13. I talked less than usual				
14. I felt lonely				
15. People were unfriendly				
16. I enjoyed life				
17. I had crying spells				
18. I felt sad				
19. I felt that people disliked me				
20. I could not get “going”				

*Below is a list of some of the ways you may have felt or behaved. Please indicate how often you have felt this way during the past week by checking the appropriate box against each question.*
